# Assessment of Benzo(a)pyrene-equivalent Carcinogenicity and Mutagenicity of Residential Indoor *versus* Outdoor Polycyclic Aromatic Hydrocarbons Exposing Young Children in New York City

**DOI:** 10.3390/ijerph7051889

**Published:** 2010-04-27

**Authors:** Kyung Hwa Jung, Beizhan Yan, Steven N. Chillrud, Frederica P. Perera, Robin Whyatt, David Camann, Patrick L. Kinney, Rachel L. Miller

**Affiliations:** 1 Division of Pulmonary, Allergy and Critical Care of Medicine, College of Physicians and Surgeons, Columbia University, PH8E, 630 W. 168 St. New York, NY 10032, USA; E-Mail: kj2237@columbia.edu; 2 Lamont-Doherty Earth Observatory, Columbia University, 61 Rt, 9W Palisades, NY 10964, USA; E-Mails: yanbz@ldeo.columbia.edu (B.-Z.Y.); chilli@ldeo.columbia.edu (S.N.C.); 3 Mailman School of Public Health, Department of Environmental Health Sciences, Columbia University, 60 Haven Ave., B-1 New York, NY 10032, USA; E-Mails: fpp1@columbia.edu (F.P.P.); rmw5@columbia.edu (R.W.); plk3@columbia.edu (P.L.K.); 4 Southwest Research Institute, 6220 Culebra Road, San Antonio, TX 78228, USA; E-Mail: david.camann@swri.edu

**Keywords:** risk assessment, PAH, BaP-equivalents, TEF, MEF, heating season, indoor, outdoor, and children

## Abstract

The application of benzo(a)pyrene (BaP)-toxic equivalent factor to polycyclic aromatic hydrocarbons (PAH) concentrations can provide a more accurate risk assessment from environmental exposure to PAH. We hypothesized that BaP-equivalent toxicity determined following residential air monitoring among young urban children may vary by season. Residential indoor and outdoor air levels of PAH measured over two-weeks in a cohort of 5–6 year old children (n = 260) in New York City were normalized to the cancer and mutagen potency equivalent factor of BaP (BaP = 1). Data are presented as carcinogenic equivalents (BaP-TEQ) and mutagenic equivalents (BaP-MEQ) for the sum of 8 PAH (Σ_8_PAH; MW ≥ 228) and individual PAH and compared across heating versus nonheating seasons. Results show that heating compared to nonheating season was associated significantly with higher (BaP-TEQ)_Σ8PAH_ and (BaP-MEQ)_Σ8PAH_ both indoors and outdoors (p < 0.001). Outdoor (BaP-TEQ)_Σ8__PAH_ and (BaP-MEQ)_Σ8PAH_ were significantly higher than the corresponding indoor measures during the heating season (p < 0.01). These findings suggest that at levels encountered in New York City air, especially during the heating season, residential exposure to PAH may pose an increased risk of cancer and mutation.

## 1. Introduction

Many polycyclic aromatic hydrocarbons (PAH) are suspected or known carcinogens and mutagens [1–3]. Benzo(a)pyrene (BaP), believed to be the most toxic PAH, has been well-characterized toxicologically. However, less information is available for most of the other PAH. In most risk assessments, many individual PAH have been considered to be of equivalent toxicity as BaP [4]. This approach could result in the overestimation of cancer and mutagen potency of individual PAH because most PAH are considerably less toxic than BaP when analyzed in the same assay systems (e.g., mouse skin, human cell mutagenicity assays) [1–3,5].

Several approaches have been developed to obtain a more accurate assessment of potential risk of exposure to a complex mixture of PAH using toxic equivalency factors based on BaP [1–3,6–8]. One is the carcinogenic equivalency factors (TEF) that can vary at either low or high dose settings. For example, TEF of dibenz(ah)anthracene (DahA) is around 5 at low dose and close to 1 at higher dose based on local tumors induced by subcutaneous injection into mice [9]. Mutagenicity of individual PAH relative to BaP also has been assessed using the mutagenic equivalency factor (MEF) proposed by Durant *et al*. [1,2]. Mutagenic activity, while not as uniformly associated with cancer [10–12], may have implications for other non-cancerous adverse health effects, such as pulmonary diseases [13,14].

TEF and MEF values in combination with measured air concentrations have been used for the calculation of carcinogenic equivalents (TEQ, expressed in ng/m^3^) and mutagenic equivalents (MEQ, expressed in ng/m^3^) in environmental samples [15–17]. To date, these studies have yielded important reassessments of the contributions of exposure to PAH, including those derived from traffic emissions, on lung cancer risk [17]. However, most pediatric cohort research that addresses the adverse health effects of exposure to air pollution, and PAH specifically, have compared levels of airborne PAH, or their metabolites, with clinical outcomes [18]. Use of the TEQ or MEQ may lead to a more accurate assessment of potential health risk in a pediatric cohort.

We hypothesized that BaP-equivalent toxicity, when assessed using residential monitoring in a pediatric urban cohort, varies by season due to higher emissions from heating sources and more frequent stagnant meteorological conditions in the winter. Our approach was to calculate BaP-TEQ and BaP-MEQ to estimate residential indoor and outdoor PAH carcinogenic and mutagenic hazards in young inner city children, known to be at greater risk for adverse health consequences from exposure to air pollution [18–20]. BaP-TEQ is based specifically on the report by Nisbet and Lagoy [3] and was determined at relatively low doses compared to other reports and may be an more appropriate method for reassessing the potential risk of airborne exposure to PAH in urban settings [3,6,7].

## 2. Experimental Section

**Study design.** Children were primarily of African-American and Dominican ethnicity and lived in Northern Manhattan and the South Bronx, geographical areas where exposure to traffic-related air pollution has been implicated in asthma and other diseases [19]. 260 children from the parent Columbia Center for Children’s Environmental Health (CCCEH) cohort study was included in this analysis [18,20] if they were age 5–6 years beginning October 2005 and resided in Northern Manhattan and the South Bronx during pregnancy and continued to live in Northern Manhattan and the Bronx at enrollment as described [21]. The study was approved by the Columbia University Institutional Review Board and informed consent obtained.

**Residential monitoring.** Boxes containing up to three vacuum pumps and valves to control flow were used to collect two-week integrated indoor and outdoor PAH samples at each of 260 homes between October 2005 and May 2009. Indoor air monitors were placed in a room where the child spent most of his or her time (e.g., child’s bedroom or main living area of the apartment), at a height of about 1.2 m and at least 0.3 m from the walls. At one third of homes, selected randomly but evenly across all 4 meteorological seasons, simultaneous outdoor sampling was conducted by placing samplers out of windows securely hung 0.9 m from the outside wall with a window unit that was designed so as not to appreciably affect air exchange rates of the apartment (*i.e.*, subject can have the window open or closed).

Particulate phase of PAH on a quartz microfiber filter was collected in a cassette attached downstream from a cyclone with a 2.5 μm aerodynamic-diameter cut point (model SCC 1.062, BGI, Inc.). Gas phases of PAH were collected on polyurethane foam (PUF) cartridge back-up, as previously described [21,22]. The residential air sampling pumps operated continuously at 1.5 L/min for two weeks, leading to an average sampling volume of 30.1 m^3^. The air flow rates were checked at the beginning and end of sampling to ensure that a constant flow rate was maintained throughout the sampling period. Eight 4-ring to 6-ring PAH were selected as target compounds due to their abundance in traffic emissions and their possible carcinogenicity and mutagenicity [23,24]. The eight PAH monitored were: benz[a]anthracene (BaA), chrysene/iso-chrysene (Chry), benzo[b]fluoranthene (BbFA), benzo[k]fluoranthene (BkFA), benzo[a]pyrene (BaP), indeno[1,2,3-c,d]pyrene (IP), dibenz[a,h]anthracene (DahA), benzo[g,h,i]perylene (BghiP). A single soxhlet extraction of both the filters and PUFs together was analyzed at Southwest Research Institute (San Antonio, TX) as described [23]. Two deuterated compounds (anthracene-d_10_ and p-terphenyl-d_14_) were used as surrogate standards for recovery and chrysene-d_12_ and perylene-d_12_ were used as internal standard for quantification.

**Calculation of BaP-equivalent concentrations.** BaP-TEQ (carcinogenic equivalents, ng/m^3^) and BaP-MEQ (mutagenic equivalents, ng/m^3^) were calculated by multiplying the concentrations of each PAH compound with its TEF for cancer potency relative to BaP [3] and MEF relative to BaP [1–2], respectively. BaP-TEQ and BaP-MEQ levels for the sum of nonvolatile PAH (∑_8_PAH; MW≥228) were calculated as follows:

- (BaP-TEQ)_Σ8PAH_ = [BaA] × 0.1 + [Chry] × 0.01 + [BbFA] × 0.1 + [BkFA] × 0.1 + [BaP] × 1 + [IP] × 0.1 + [DahA] × 5 + [BghiP] × 0.01.
- (BaP-MEQ)_Σ8PAH_ = [BaA] × 0.082 + [Chry] × 0.017 + [BbFA] × 0.25 + [BkFA] × 0.11 + [BaP] × 1 + [IP] × 0.31 + [DahA] × 0.29 + [BghiP] × 0.19.

**Quality control.** Each air monitoring result was assessed and flagged if there are any issues of sampling conditions such as tube disconnection from the pump, late-takedown, pump failure, switch error, and any other human errors. Once flagged, air monitoring data were given a quality assurance (QA) score of 1 (0: highest quality) and further examined for additional score for erroneous length of sampling time, erroneous flow rate of pump, and missing documentation. If a final QA score is ≥ 3, the data was excluded, sampling was redone. Flagged data were included for analysis if they passed a quality control test (QA ≤ 2), as described [25]. Five failed the quality control test.

Mean recovery of deuterated surrogate standards was 97.9% (±17% Standard deviation, SD) and 102.6% (±15%, SD) for d10 anthracene and d14-p-terphenyl, respectively in all batches except for one. In one batch of measures, the mean recovery efficiency exceeded 130% in some samples (attributed to evaporation during storage) and adjustment was made downward by the multiplier 100/ (mean recovery) and included for the data analysis. The limit of detection (LODs) for 8 individual PAH was 0.03 ng/m^3^.

**Statistical analysis.** Descriptive statistics were used to describe overall BaP-TEQ and BaP-MEQ concentrations for individual PAH and ∑_8_PAH indoors and outdoors. Due to the non-normal distributions of individual PAH and the sum of 8 PAH (∑_8_PAH) concentrations, Mann-Whitney and Wilcoxon signed ranks test were conducted. Heating season was defined as any sampling that was initiated October 1st through April 30st as described [21]. Indoor and outdoor comparisons were based on the subset of homes in which both indoor and outdoor air concentrations were measured simultaneously. Analyses were conducted using SPSS software (SPSS; Chicago, IL, version 17).

## 3. Results

### Residential indoor and outdoor BaP-equivalents

The BaP-equivalent (BaP-TEQ and BaP-MEQ) concentrations calculated for Σ_8_PAH and individual PAH measured in this study are shown in Table 1. The levels of indoor (BaP-TEQ)_Σ8PAH_ and (BaP-MEQ)_Σ8PAH_ ranged from 0.098–8.348 ng/m^3^ and 0.069–19.72 ng/m^3^, respectively. For all samples studied, the largest contribution of individual PAH to (BaP-TEQ)_Σ8PAH_ and (BaP-MEQ)_Σ8PAH_ was made by BaP, followed by DahA for (BaP-TEQ)_Σ8PAH_ and IP and BghiP for (BaP-MEQ)_Σ8PAH_ (Table 1). In outdoor air, the contribution of BbFA to (BaP-MEQ)_Σ8PAH_ was substantially elevated when compared to indoor air, making it the dominant compound contributing to (BaP-MEQ)_Σ8PAH_.

### Seasonal variations in BaP-equivalents

**Figure 1.**
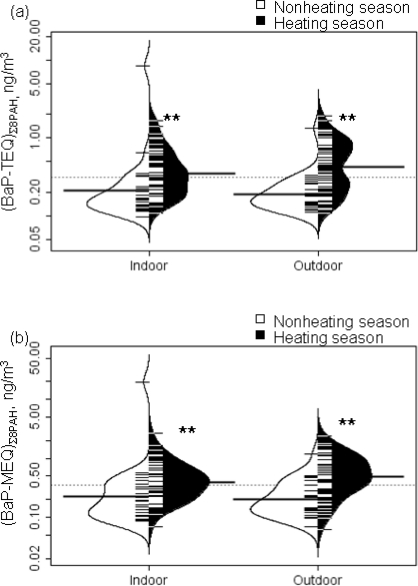
Seasonal variations in **(a)** (BaP-TEQ)_∑8PAH_ and **(b)** (BaP-MEQ)_∑8PAH_. Note: Mann-Whitney test was performed to compare heating season and nonheating concentrations (BaP-TEQ)_∑8PAH_ and (BaP-MEQ)_∑8PAH_ indoors and outdoors. The white and black lines show individual observations, while the white and black area shows the distribution. The dotted line indicates the overall geometric mean and the thicker solid line shows the geometric mean concentration of indoors and outdoors for each season. Mann-Whitney test, **p<0.001. ∑_8_PAH includes benz(a)anthracene (BaA), chrysene/iso-chrysene (Chry), benzo(b)fluoranthene (BbFA), benzo(k)fluoranthene (BkFA), benzo(a)pyrene (BaP), indeno(1,2,3-c,d)pyrene (IP), dibenz(a,h)anthracene(DahA), and benzo(ghi)perylene (BghiP).

Heating compared to nonheating season was associated significantly with higher (BaP-TEQ)_Σ8PAH_ and (BaP-MEQ)_Σ8PAH_ both indoors and outdoors (Figure 1-a and Figure 1-b; p < 0.001, Mann-Whitney test). This pattern was apparent when the individual 8 PAH were assessed (p < 0.001).

In addition, during the heating season, outdoor (BaP-TEQ)_Σ8PAH_ and (BaP-MEQ)_Σ8PAH_ were significantly higher than the corresponding indoor measures (Table 2; p < 0.01, Wilcoxon signed ranks test). A similar pattern was not observed during the nonheating season (Table 2; p > 0.05, Wilcoxon signed ranks test). Similarly, most individual 8 PAH (except BaP and IP) were higher outdoors compared to indoors during the heating season (p < 0.05). BbFA and Chry were significantly higher outdoors than indoors regardless of season (p < 0.05, Wilcoxon signed ranks test).

## 4. Discussion and Conclusions

Our objective was to estimate the potential carcinogenic and mutagenic risks of residential exposure to PAH for a cohort of inner city young children based on BaP-equivalent concentration (BaP-TEQ and BaP-MEQ). We found a significant effect of heating season on BaP-TEQ and BaP-MEQ possibly due to higher emissions from heating sources and more frequent stagnant meteorological conditions in the winter. While an effect of heating season has been documented in small studies [15,16], this is the first paper to examine seasonal differences in carcinogenic and mutagenic risks based on residential exposure of a large cohort of urban children.

The indoor (BaP-TEQ)_Σ8PAH_ levels observed in this study were considerably lower than those reported for other homes impacted heavily by industrial and traffic emissions in Ohura *et al.* [15]. (BaP-TEQ)_Σ8PAH_ risk levels depend not only on concentrations of individual 8 PAH, but also the composition of PAH mixtures affected by varying emission sources. While ∑_8_PAH concentrations measured in the Ohura study were 1.3–2.2 times higher than those measured in this study, (BaP-TEQ)_Σ8PAH_ levels were 2–3.5 times higher. Consistent with Ohura *et al*. [15], we observed that BaP was the dominant compound contributing to (BaP-TEQ)_Σ8PAH_, accounting for 45% of indoor (BaP-TEQ)_Σ8PAH_ and 35% of outdoor (BaP-TEQ)_Σ8PAH_.

While the contributions of the sum of IP and BghiP to (BaP-TEQ)_Σ8PAH_ both indoors and outdoors were only 11–12%, their contributions to (BaP-MEQ)_Σ8PAH_ were much higher considerably (45–48%). Considering that these compounds are considered tracers of vehicular emissions [26], exposure to traffic emissions may impact the mutagenicity risk to a greater extent than the carcinogenic risk.

Both indoors and outdoors, higher (BaP-TEQ)_Σ8PAH_ and (BaP-MEQ)_Σ8PAH_ risks were observed in the heating season, compared to the nonheating season. The heating season may be associated with (1) increased use of fossil fuel combustion for residential heating (2) reduced air exchange rates (AERs) [27], (3) reduced PAH transformation through photochemical/chemical reaction due to a lower temperature and ozone concentration [28], (4) gas/particle partitioning in favor of the particulate phase with lower temperature and (5) frequent stagnant meteorological conditions such as a lower mixing height. Presumably some combination of these characteristics of heating season led to changes in either concentrations or relative composition that was pertinent to the calculation of these measures.

Several studies reported that heavier 5–7 ring PAH have higher airborne measures outdoors compared to indoors, due to the presence of major outdoor emission sources (*i.e.*, traffic sources, industry, and power generation etc) of those compounds [15,29]. Heavier PAH concentrations indoors are strongly affected not only by the outdoor concentrations but also by outdoor-to-indoor AERs that were shown to be lower during winter than summer when residential windows usually are shut [15,27,29]. Similar trends were obtained for (BaP-TEQ)_Σ8PAH_ and (BaP-MEQ)_Σ8PAH_ during the heating season. This result suggests that children are subjected to higher carcinogenic and mutagenic risks derived from PAH when they play outside more so than inside the home during the heating season. When compared to other urban outdoor values measured in the winter season [15–17], outdoor (BaP-TEQ)_Σ8PAH_ and (BaP-MEQ)_Σ8PAH_ observed in this study were 2–5 times lower during the heating season.

These risks measured and calculated here raise significant concerns for public health. For example, applying the World Health Organization (WHO) suggested unit risk of 8.7 × 10^−5^ (ng/m^3^)^−1^ for lifetime (70 years) PAH exposure [30], the corresponding lifetime lung cancer risks were found to be 4.2 × 10^−5^ (= 0.478 ng/m^3^ × 8.7 × 10^−5^ (ng/m^3^)^−1^) and 5.1 × 10^−5^ (= 0.590 ng/m^3^ × 8.7 × 10^−5^ (ng/m^3^)^−1^) when the average indoor (BaP-TEQ)_Σ8PAH_ and (BaP-MEQ)_Σ8PAH_ concentration were used, respectively. Hence, if 1,000,000 people were exposed to 0.478 ng/m^3^ of indoor (BaP-TEQ)_Σ8PAH_ for 70 years, then 42 people may develop lung cancer. An excess lung cancer risk from lifetime exposure to (BaP-MEQ)_Σ8PAH_ concentration would be 51 cases among one million individuals exposed. The estimated cancer risks from air pollution in NYC did not exceed a health-based guideline (8.7 × 10^−5^) calculated based on the maximum permissible level of 1 ng/m^3^ of BaP [30]. However, it should be noted that the risk estimates presented are very uncertain, and could be understood only as a crude estimation of cancer risk from the PAH inhalation.

We acknowledge study limitations. The potential risk of PAH exposure based on TEQ or MEQ may be underestimated if the interaction of some PAHs are synergistic rather than additive. Chemical degradation of PAH by ambient oxidants (ozone, hydroxyl radical, or nitrogen oxides) in the atmosphere as well as on the filters during sampling could also underestimate the potential risks due to reduced measured PAH levels, as shown in several studies [28,31–36]. For example, BaP concentrations collected without denuder that can remove atmospheric oxidants such as ozone and OH radical can be underestimated by more than 200% of the measured value at high ozone levels in summer [28,31]. Also PAH collected on the filters may be decomposed through heterogeneous chemical reactions with ozone during an extended sampling period [28,34–36]. Furthermore, nitrated/oxygenated PAH compounds have not been measured in this study, underestimating the full carcinogenic and mutagenic potential of PAH exposure. Those PAH compounds formed by photochemical/chemical reactions are known to be more toxic than their parent PAH based on tumorigenicity of PAH in a newborn mouse assay [8,37,38]. Young children can be exposed to PAH through other routes besides inhalation. These include ingesting food, nondietary ingestion of dust or soil through hand-to-mouth activity, or dermal contact with soil polluted by PAH [39,40]. Although inhalation is an important pathway for inner-city children because of high levels of PAH measured in indoor and outdoor air, dietary ingestion and non-dietary ingestion pathways are thought to be more important for young children’s exposure to heavier PAHs [39,40]. Thus, the values reported in this study may need to be considered as the lower limit of estimated potential PAH health risk resulted from inhalation of air. Further investigations are needed whether BaP-equivalent levels are associated with any observed health outcomes (*i.e.*, respiratory or allergic symptoms *etc*.) within the cohort.

In conclusion, we found that heating season is an important contributor to the potential risk of PAH exposure. This finding has implications for the design of environmental health studies that focus on air pollution exposure and young children living in the inner city.

## Figures and Tables

**Table 1.** BaP-equivalent carcinogenicity and mutagenicity risks assessed in 5–6 year old residential indoor and outdoor air.

		**BaP-TEQ Levels, ng/m^3^**					**BaP-MEQ Levels, ng/m^3^**				
**Analyte**	**n**	**aTEF**	**Median**	**Mean**	**SD**	**Range**	**bMEF**	**Median**	**Mean**	**SD**	**Range**
Σ_8_PAH	255	NA	0.299	0.478	0.709	0.098–8.348	NA	0.376	0.590	1.325	0.069–19.72
BaP	255	1	0.121	0.198	0.363	0.015–4.494	1	0.121	0.198	0.363	0.015–4.494
BaA	255	0.1	0.006	0.009	0.010	0.002–0.132	0.082	0.005	0.007	0.009	0.001–0.108
Chry	255	0.01	0.001	0.001	0.002	0.000–0.021	0.017	0.001	0.002	0.003	0.000–0.036
BbFA	255	0.1	0.019	0.028	0.034	0.004–0.346	0.25	0.047	0.069	0.084	0.010–0.865
BkFA	255	0.1	0.006	0.010	0.013	0.002–0.140	0.11	0.007	0.010	0.014	0.002–0.154
IP	255	0.1	0.028	0.044	0.094	0.002–1.400	0.31	0.087	0.136	0.291	0.006–4.340
DahA	255	5	0.083	0.180	0.212	0.055–1.741	0.29	0.005	0.010	0.012	0.003–0.101
BghiP	255	0.01	0.004	0.008	0.033	0.001–0.517	0.19	0.084	0.156	0.624	0.016–9.828
**Outdoor**											
Σ_8_PAH	82	NA	0.277	0.450	0.389	0.109–1.932	NA	0.360	0.528	0.454	0.062–2.394
BaP	82	1	0.091	0.133	0.133	0.016–0.748	1	0.091	0.133	0.133	0.016–0.748
BaA	82	0.1	0.007	0.011	0.011	0.001–0.059	0.082	0.005	0.009	0.009	0.001–0.048
Chry	82	0.01	0.001	0.002	0.002	0.0003–0.008	0.017	0.002	0.004	0.003	0.001–0.013
BbFA	82	0.1	0.039	0.048	0.043	0.006–0.237	0.25	0.097	0.121	0.108	0.015–0.592
BkFA	82	0.1	0.010	0.015	0.017	0.002–0.114	0.11	0.011	0.017	0.018	0.002–0.125
IP	82	0.1	0.027	0.035	0.031	0.003–0.197	0.31	0.085	0.107	0.095	0.010–0.611
DahA	82	5	0.083	0.199	0.188	0.075–1.021	0.29	0.005	0.012	0.011	0.004–0.059
BghiP	82	0.01	0.005	0.007	0.007	0.001–0.039	0.19	0.087	0.126	0.126	0.010–0.739

a TEF: toxic equivalency factors for cancer potency relative to BaP (Nisbet and LaGoy, 1992)

b MEF: mutagenic potency factor relative to BaP (Durant *et al*., 1996 and 1999)

BaP-TEQ: Carcinogenic equivalents calculated from the cancer potency relative to BaP (TEF) multiplied by the concentration of PAH in a sample.

BaP-MEQ: Mutagenic equivalents calculated from the mutagenic potency relative to BaP (MEF) multiplied by the concentration of PAH in a sample.

∑_8_PAH includes benzo(a)pyrene (BaP), benz(a)anthracene (BaA), chrysene/iso-chrysene (Chry), benzo(b)fluoranthene (BbFA), benzo(k)fluoranthene (BkFA), indeno(1,2,3-c,d)pyrene (IP), dibenz(a,h)anthracene(DahA), and benzo(ghi)perylene(BghiP).

**Table 2.** Relationship between indoor and outdoor BaP-equivalent carcinogenicity and mutagenicity risks, stratified by season.

**Season**	**Measure**	**Indoor, ng/m^3^** (n^a^ = 55; n^b^ = 26)		**Outdoor, ng/m^3^** (n^a^ = 55; n^b^ = 26)		
		Average ± SD	Median	Average ± SD	Median	p
Heating	(BaP-TEQ)_∑8PAH_	0.460 ± 0.365	0.346	0.558 ± 0.407	0.485	0.002**
	(BaP-MEQ)_∑8PAH_	0.521 ± 0.448	0.388	0.660 ± 0.480	0.500	0.001**
Nonheating	(BaP-TEQ)_∑8PAH_	0.515 ± 1.602	0.163	0.232 ± 0.233	0.167	0.989
	(BaP-MEQ)_∑8PAH_	0.975 ± 3.826	0.174	0.260 ± 0.230	0.173	0.989

** (p<0.01) for Wilcoxon Signed Ranks test.

n^a^: Number for heating season,

n^b^: Number for nonheating season

Heating season was defined as any sampling that was initiated October 1^st^ through April 30^st^.
